# Use of factor scores in multiple regression analysis for estimation of body weight by certain body measurements in Romanov Lambs

**DOI:** 10.7717/peerj.7434

**Published:** 2019-08-01

**Authors:** Yalcin Tahtali

**Affiliations:** Faculty of Agriculture, Department of Animal Science, Gaziosmanpasa University, Tokat, Turkey

**Keywords:** Live weight, Regression, Factor analysis, Romanov lambs

## Abstract

The study investigates the solution of the multicollinearity between certain body measurements of Romanov lambs and prediction of the body weight of Romanov lambs using the thus calculated factor analysis scores and a multiple regression model. For this purpose, the body measurements (wither height (WH), croup height (CH), body length (BL), chest depth (CD), chest circumference (CC), chest width behind shoulders (CWS) and head length (HL)) and body weight (BW) of 6-month-old 50 Romanov lambs born in 2015 were used. The factor analysis scores were used to obtain the prediction equation for the relationship between the investigated traits. The analysis results showed that there was a multicollinearity between the wither and croup height traits used in the prediction equation. Moreover, the results revealed that the variables for the body measurements can be represented by two factors. These factors explained 50.89% and 22.86% of the total variance, respectively. The multicollinearity between the independent variables was eliminated with the use of the factor scores obtained with the factor analysis in the multiple regression model, and thus it was observed that better results can be obtained by using the factor analysis scores in the prediction of the body weight of 6-month-old Romanov lambs.

## Introduction

In animal husbandry, knowing the relationship between body weight and other body measurements is of great importance. These measurements are related both to genetic and environmental conditions and used as the indirect selection criteria in the use of animals in future generations. Thus, prior knowledge about these relationships give important information in the evaluation of the animals for early selection (Cankaya et al., 2009).

The multiple regression model is the most commonly used prediction model for the interpretation of the relationship between body weight and certain body measurements and prediction equations are usually obtained using the least squares method without taking the relationship between independent variables into account (Draper & Smith, 1981; Cankaya et al., 2006; Sangun et al., 2009). However, the method, which is used to predict the body weight of animals, has certain drawbacks. Significant multicollinearity between independent variables may lead to some inaccuracies in the statistical interpretation of the coefficients of the regression parameters estimated by least squares method. One way to avoid this problem is the use of the factor scores, which are obtained with factor analysis and independent from each other, i.e., orthogonal factors, in regression analysis instead of the direct application of the least squares method to the original data set. For multiple regression models, factor analysis scores can be employed along with correlation analysis to solve the multicollinearity problem. With the use of the scores, the multicollinearity problem between independent variables (body measurements) can be solved (Tabachnick & Fidell, 2001; Keskin, Daskiran & Kor, 2007).

Factor analysis aims to identify the structure underlying a data matrix and, in addition to its basic functions, is of importance in the application of certain multivariate statistical methods. In its general sense, factor analysis is a set of methods explaining a structure that is explained with *p* number of correlated variables with a smaller number (*k < p*) of new variables (factor) that are related on their own but not related to each other (Kleinbaum et al., 1998). In addition, it aims to bring out smaller number of new variables (factors), which are significant in terms of independent terms, from multiple variables that are difficult to interpret and correlated with each other with minimum information loss. Factor analyses are used to eliminate the variable.

The study investigates the usability of the factor analysis scores in the prediction of various body measurements (wither and croup heights, body length, chest width and circumference, chest width behind shoulders and head length) of Romanov lambs with multiple regression. Moreover, the number of studies on the use of factor scores in the multiple regression analysis for the prediction of various body measurements of Romanov lamb is limited.

## Material and Method

The study material comprised 50 Romanov lambs that were brought to Gürçeşme Village of Niksar Town in Tokat, Turkey, from Nikopol, Ukraine, on 15.07.2015. The lambs were fed with the same ration program beginning from the weaning period (day 75) and until the end of the trial (day 180). The lambs were not grazed during the trial period. Before the weaning period, each lamb was individually monitored and an average of 200–300 gr, 2,500 kcal/ME and 12–16% HP-containing lamb starter concentrate feed was fed to the lambs by taking their body weights and how much they suckle from their mothers into account. Water was kept in a constantly accessible location. After the weaning period, the lambs were separated from their mothers and divided into specific groups based on their birth dates. Again, the rations programs were used and 2,500 kcal/ME and 18–21% HP-containing concentrate feed and high-quality crude feed were fed to the lambs by taking their body weights and ages into account. The feeding programs were applied by carefully and homogenously calculating the feed amounts.

In the study, the body weight (BW), wither-height (WH), croup-height (CH), body length (BL), chest depth (CD), chest circumference (CC), chest width behind shoulders (CWS) and head length (HL) of the lambs born at different dates were recorded at birth and 15 days after birth and every 15 days until month 6. Among these traits, the body measurement on month 6 constituted the independent variables (X variable set), while the measurements for body weight constituted the dependent variable (Y variable).

The multiple regression analysis was used in the study is one of the methods to describe the relationship between one dependent variable and multiple independent variables. Equation (1) is the general expression of the multiple regression model in the multiplicative form.

(1) \begin{eqnarray*}& & {Y}_{i}={\beta }_{0}{X}_{{i}_{1}}^{{\beta }_{1}}{X}_{{i}_{2}}^{{\beta }_{2}}{X}_{{i}_{3}}^{{\beta }_{3}}\ldots {X}_{{i}_{p}}^{{\beta }_{p}}{e}_{i};i=1,2,3,\ldots ,n.\end{eqnarray*}

In the equation,

*β*_*j*_ represents the regression parameters; *j=1,2,3,…, p*

*e*_*i*_ represents the error term with a mean of 0, variance of *σ*^2^ andnormal distribution

*Y*_*i*_ represents the dependent variable of the study

*X*_*i*1_, *X*_*i*2_, *X*_*i*3_, …, *X*_*ip*_ represent the independent variables of the study.

The data does not always show a normal distribution. When the data are shown on a graph, the curve is not always linear. In other words, the relationship between the traits can show a curved distribution. Logarithmic transformation is applied to the *X* and *Y* observed variables to linearize the curvature. In this case, Eq. (1) transforms into the models given in Eqs. (2) and (3).

(2) \begin{eqnarray*}ln{Y}_{i}=ln{\beta }_{0}+{\beta }_{1}ln{X}_{i1}+\ldots +{\beta }_{p}ln{X}_{ip}+ln{e}_{i}\end{eqnarray*}

Or by taking ${b}_{1}={\hat {\beta }}_{1},\ldots ,{b}_{p}={\hat {\beta }}_{p}$,

(3) \begin{eqnarray*}{\hat {Y}}_{i}=a+{b}_{1}{z}_{i1}+{b}_{2}{z}_{i2}+{b}_{3}{z}_{i3}+\ldots +{b}_{p}{z}_{ip}.\end{eqnarray*}

In the above equations, *Y* = *lnY*_*i*_ represents the body weights; by taking (*p* =1,2,…7),

*z*_*i*1_ = *lnX*_*i*1_, *z*_*i*2_ = *lnX*_*i*2_, …, *z*_*ip*_ = *lnX*_*ip*_,  represents the independent variables (wither and croup heights, body length, chest depth and circumference, chest width behind shoulders, head length); *β*_1_, *β*_2_, …, *β*_*p*_ and $a=ln \left( {\beta }_{0} \right) ,$ represent the regression parameters; $ln \left( {e}_{i} \right) $ represents the random error, respectively (Gunst & Mason, 1980; Draper & Smith, 1981; Kleinbaum et al., 1998).

The *t*-test statistics given in Eq. (4) are used to test the statistical significance of the multiple regression coefficients that are estimated using the multiple regression analysis.

(4) \begin{eqnarray*}& & {t}_{j}= \frac{{b}_{j}-{\beta }_{j}}{\sqrt{var({b}_{j})}} \sim {t}_{\alpha (n-p-1)};j=1,2,3,\ldots ,p.\end{eqnarray*}

In the above equation,

*b*_*j*_ represents the regression coefficients estimated with the least squares method,

$var \left( {b}_{j} \right) $ represents the variance of the estimated regression coefficients,

(*n* − *p* − 1) represents the degrees of freedom,

*n* represents the sample size,

*p* represents the number of variables and

*α* represents the type I error.

In multiple regression analysis, one of the assumptions for interpretation with the least squared method is the lack of a significant relationship between the independent variables (absence of multicollinearity): *Cov*(*X*_*i*_, *X*_*j*_) = 0; i≠ j. However, multicollinearity can arise between the independent variables that are used to predict the dependent variable in the analysis results. Collinearity is defined as the presence of a complete or high-degree correlation between two or more explanatory (independent) variables in multiple regression analysis. In the case of multicollinearity, the evaluation of the effect of independent variables on the dependent variables is challenging (Eyduran, Topal & Sonmez, 2010). In other words, in the presence of multicollinearity, the variances and covariances of the regression coefficients increase and regardless of a high *R*^2^ value, only a small portion of the independent variables turns out significant according to the *t*-test (Gujarati, 1995).

Such cases can result in eliminating the wrong variable from the model and incorrectly specifying the model (specification error). Thus, the variance inflation factors (VIF) given in Eq. (5) should be calculated to reveal the multicollinearity.

(5) \begin{eqnarray*}VIF= \frac{1}{(1-{R}^{2})} .\end{eqnarray*}

To interpret the presence of multicollinearity in the analyses, the calculated *R*^2^ value should be high and the calculated VIF value should be 10 or greater, which indicatethe presence of a multicollinearity problem (Johnson & Wichern, 2002).

In the multiple regression analysis applications, an estimation method based on the factor scores that were estimated from the factor analysis can be used to eliminate the limitations caused by the multicollinearity problem between the independent variables. The main purpose of factor analysis is to allow understanding and interpreting the supposed relationship between multiple variables and representing multiple variables with a smaller number of factors (Tinsley & Brown, 2000).

The factor equation is given in Eq. (6) in its matrix form.

(6) \begin{eqnarray*}\mathbi{Z}=\lambda F+\varepsilon .\end{eqnarray*}

In the equation;

*Z* represents the vector of the *px1*-dimensional variable,

*λ* represents the matrix of the *pxm*-dimensional factor loads,

*F* represents the *mx1*-dimensional factor vector,

ε represents the *px1*-dimensional error vector (Sharma, 1996).

In factor analysis, the Bartlett’s test and Kaiser–Meyer-Olkin (KMO) test are applied to test the divisibility of the correlation matrix into factors. If the null hypothesis is rejected according to results of the Bartlett’s test, the factor analysis is continued (Sharma, 1996). Obtaining a value below 0.5 with the KMO test indicates that the relationship between the variable pairs cannot be explained by other variables (Celik et al., 2018).

Some researchers have reported that KMO values of about 0.6, 0.7, 0.8 and 0.9 are mediocre, middling, meritorious and marvelous, respectively (Sharma, 1996; Karagoz & Kosterelioglu, 2008).

In factor analysis, the eigenvalues are obtained from the correlation matrix. In the study, for the interpretation of the factor loads, the varimax rotation was employed and factor coefficients were used to obtain the factor scores for the selected factor (Cankaya et al., 2009; Keskin, Daskiran & Kor, 2007).

Hence, by deriving orthogonal (independent) factor scores and with the use of these coefficients, the multicollinearity problem between the independent factors that are used to predict body weight is solved. In the analysis results, the number of the factors used in the multiple regression model is usually represented by the number of the eigenvalues that are greater than 1, which are obtained from the correlation matrix (Sharma, 1996; Tinsley & Brown, 2000).

In the study, all statistical calculations for the prediction of the body weights of the Romanov lambs using the body measurements of the lambs measured on day 180 were performed using the SPSS 21.0 statistical package program.

## Results

Table 1 shows the descriptive statistics for the body weight and certain body measurements of 50 Romanov lambs born in 2015 in a husbandry in Niksar Townin Tokat, Turkey, that were measured on day 180. The statistical analyses showed that the investigated traits showed normal distribution according to the Kolmogorov–Smirnov normality test (*P* > 0.05).

**Table 1:** Descriptive statistics for the morphological traits by body measurements.

Parameter	*n*	Mean ± Std. Error
Body weight (BW)	50	25.067 ± 0.355
Croup height (CH)	50	54.772 ± 0.398
Wither height (WH)	50	54.386 ± 0.408
Body length (BL)	50	50.316 ± 0.322
Chest depth (CD)	50	25.484 ± 0.208
Chest circumference (CC)	50	74.502 ± 0.385
Chest width behind shoulders (CWS)	50	13.260 ± 0.111
Head length (HL)	50	13.828 ± 0.140

Table 2 shows the Pearson correlation coefficients and significance tests for the body weight and body measurements of the Romanov lambs measured on day 180. As seen in the table, there was a positive relationship between the body weights and body measurements. The highest correlation was between the wither-height and croup-height (*r* = 0.993; *P* < 0.01), while the lowest correlation was between the chest depth and chest width behind shoulders (*r* = 0.179, *P* < 0.05).

**Table 2:** Pearson correlation coefficient between the morphological traits.

	BW	CH	WH	BL	CD	CC	CWS
CH	0.860^**^						
WH	0.840^**^	0.993^**^					
BL	0.655^**^	0.698^**^	0.690^**^				
CD	0.513^**^	0.528^**^	0.535^**^	0.445^**^			
CC	0.780^**^	0.709^**^	0.706^**^	0.671^**^	0.586^**^		
CWS	0.408^**^	0.336^*^	0.339^*^	0.257	0.179	0.284^*^	
HL	0.317^*^	0.317^*^	0.323^*^	0.328^*^	0.281^*^	0.216	0.502^**^

**Notes.**

* *P* < 0.05

** *P* < 0.01

The presence of a high correlation between the variables when the multiple regression analysis is used to analyze a data set (as the case in the relationship between the wither-height and croup-height) indicates that the presence of a multicollinearity problem. This can reduce the reliability of the results obtained with the least squares method. In the study, to analyze the multicollinearity problem, the prediction coefficient, standard error, test statistics and VIF values of each parameter in the multiple regression analysis were investigated and given in Table 3.

**Table 3:** Regression analysis results according to the least squares method.

Traits	Coefficients	Std.Error	*t*-value	*P*	VIF
(Constant)	−32.571	4.878	−6.676	0.000	
CH	1.463	0.487	3.002	0.005	72.067
WH	−0.922	0.473	−1.948	0.058	71.160
BL	−0.028	0.108	−0.263	0.794	2.311
CD	−0.005	0.142	−0.032	0.975	1.661
CC	0.318	0.097	3.282	0.002	2.666
CWS	0.372	0.248	1.503	0.140	1.436
HL	0.041	0.199	0.206	0.838	1.493

**Notes.**

*S* = 1.13 *R*^2^ = 82.6% R^2^ (Adjusted) = 79.7%.

The investigation of the regression analysis results according to the least square method showed that, among the body measurements used in the prediction of body weight, wither height, body length, chest depth, chest width behind shoulders and head length were statistically not significant. In addition, a multicollinearity between croup weight and wither height (VIF > 10) was observed. To solve the multicollinearity problem and for a reliable regression analysis, the factor scores obtained from the factor analysis for multiple regression analysis should be used as independent variables.

Kaiser–Meyer–Olkin (KMO) and Bartlett’s test are prerequisites for factor analysis. In other words, obtaining significant results from both tests shows that the data are suitable for factor analysis. Considering the Bartlett’s test value (309.14; *P* < 0.01) and KMO value (0.778) obtained to test the divisibility of the correlation matrix into factors, the data was determined to be suitable for factor analysis (Sharma, 1996).

Table 4 shows the results of the factor analysis. The results revealed that the eigenvalues of the first two of seven factors were greater than 1. Thus, the results showed that the first two factors were suitable for use as independent variables in the regression analysis (Keskin, Daskiran & Kor, 2007; Cankaya et al., 2009; Eyduran, Topal & Sonmez, 2010). The selected first two factors explained 50.89% and 22.86% of the total variance in all variables, adding to a total of 73.75%, respectively. On the other hand, the separate investigation of the factors revealed that the first factor explained 68.99% ((3.56/5.16) *100) of the variance, while the second factor explained 31.01% of the variance. The factor loads given in Table 4 shows the relationship between the investigated independent variables and factors. Table 4 also shows the relationship between the factor loads of the independent variables and factors. The bold values in the table shows the highest correlation between the investigated traits and factors. As a result of the analysis, factor loads of the independent variables for the first factor are cidago height (0.907), rump height (0.905), body length (0.860), chest depth (0.799) and chest circumference (0.697). In addition, the chest width behind shoulders (0.850) and the head length (0.848) were determined in the second factor.

**Table 4:** Factor analysis results.

	Factor score coefficients (c_ik_)		Rotated factor loadings (l_ik_) and communalities		
Variables	Factor 1	Factor 2	Factor 1	Factor 2	Communality
CH	0.920	−0.148	**0.907**	0.217	0.87
WH	0.920	−0.153	**0.905**	0.222	0.87
BL	0.833	−0.237	**0.860**	0.106	0.68
CD	0.812	−0.133	**0.799**	0.194	0.50
CC	0.686	−0.166	**0.697**	0.115	0.75
CWS	0.480	0.720	0.162	**0.850**	0.74
HL	0.491	0.713	0.175	**0.848**	0.75
Variance			3.56	1.60	5.16
Variance%			50.89	22.86	73.75

Moreover, the factor score coefficients of the first two factors obtained with the factor analysis were used as the independent variables in the prediction of the body weights of the Romanov lambs and the results were given in Table 5 to determine the significant factors in the prediction of the body weight.

**Table 5:** Regression analysis results according to the factor analysis results.

	Coefficients	Std.Error	*t*-value	*P*	VIF
Constant (b_0_)	25.068	0.180	139.315	<0.001	
Factor 1	2.077	0.182	11.428	<0.001	1.0
Factor 2	.663	0.182	3.650	<0.001	1.0

**Notes.**

S:1.27 *R*^2^ = 75.4% R^2^ (Adjusted) = 74.3%.

As seen in Table 5, according to the regression analysis results based on the factor analysis scores, the effects of all factors that were used as the independent variables in the prediction of the body weight of the Romanov lambs were statistically significant (*P* < 0.001).

In the model, the multicollinearity between the original independent variables that are shown in Table 3 was eliminated using the factor scores. Moreover, the factor scores used in the model explained 75.4% of the total variance of the body weights of the lambs.

## Discussion

The study was carried out both to predict the body weight using the factor analysis scores that were calculated using certain body measurements of the Romanov lambs and multiple regression model and to solve the multicollinearity between the relevant body measurements. Similar studies were also carried out on sheep and goat breeding (Keskin, Daskiran & Kor, 2007; Cankaya et al., 2006; Cankaya et al., 2009; Onk, Sarı & Gurcan, 2018; Eyduran, Karakus & Karakus, 2009; Khan et al., 2014; Yakubu, 2009; Daskiran, Keskin & Bingol, 2017; Merkhan, 2014), poultry breeding (Celik et al., 2018; Pimentel et al., 2007; Ogah, Alaga & Momah, 2009) and aquaculture (Eyduran, Topal & Sonmez, 2010; Sangun et al., 2009).

In the study, the correlation structure between the variables was primarily investigated and the correlation between the variables was determined to be high. Since the high correlation between the variables indicates the presence of multicollinearity, the prediction coefficient of each parameter in the regression analysis was investigated using the least squares method and the VIF values were investigated using the statistics. The analysis revealed a multicollinearity between croup height and wither height (VIF > 10). Furthermore, according to the least squares method, a *R*^2^ value of 82.6% and an adjusted-*R*^2^ value of 82.6% were determined in the regression analysis. The results were close to the results obtained in other studies (Cankaya et al., 2009; Onk, Sarı & Gurcan, 2018; Eyduran, Karakus & Karakus, 2009; Topal & Macit, 2004). To test the divisibility of the correlation matrix into the factors, the Bartlett’s test value (309.14; *P* < 0.01) and a KMO value of 0.778 were determined, which revealed that the data were suitable for factor analysis. Although the results were lower than the results obtained by Eyduran, Karakus & Karakus (2009) in their study investigating the relationship between body weight and body measurements, they were close to the results obtained by Khan et al. (2014) and Cankaya et al. (2009).

The factor analysis showed that the eigenvalues of the first two factors obtained from the seven independent variables were greater than 1, which led to the conclusion that the first two factors were suitable for use as the independent variables in the regression analysis. The selected first two factors explained 50.89% and 22.86% of the total variance, adding to a total of 73.75%,respectively. In their study, Keskin, Daskiran & Kor (2007) used similar variables with a three-factor structure in the regression analysis. The results were close to the results obtained in other studies (Khan et al., 2014; Daskiran, Keskin & Bingol, 2017; Keskin, Daskiran & Kor, 2007; Yakubu, 2009). Furthermore, in their study on Karayaka lambs, Cankaya et al. (2009) explained similar variables with a five-factor structureand reported that they explained 25.7%,13.6%, 13.1%,13.1%, 12.1% of the total variance, respectively. At the end of the analysis, the factor loads of the independent variables in the first two factors were determined between the wither height (0.907), croup height (0.905), body length (0.860), chest depth (0.799) and chest circumference (0.697) and factor 1 and between chest width behind shoulders (0.850) and head length (0.848) and factor 2, respectively.

The factor score coefficients of the first two factors were used as the independent variables in the prediction of the body weight of the Romanov lambs and the effects of all factors were determined to be significant (*P* < 0.001). The factor scores used for regression analysis explained 75.4% of the total variance of the Romanov lambs. Cankaya et al. (2009) determined a *R*^2^ value of 73.1% in Karayaka lambs, while Onk, Sarı & Gurcan (2018) determined a *R*^2^ value of 84.8% in the female Tuj lambs and 78.9% in the male Tuj lambs.

## Conclusion

The results of the study showed that there was a multicollinearity between the body measurements of Romanov lambs, which were used in the prediction of the body weights of 6-month-old lambs. Thus, instead of directly using these variables, the use of the factor analysis scores obtained from the variables reduced the risk of inaccurate interpretation of the parameters in the modelaccording to the least squares method.

Furthermore, using comparison, the study showed the applicability of the regression analysis results by using the classical, least squares method-based multiple regression analysis and factor analysis scores in the case of multicollinearity between independent variables.

##  Supplemental Information

10.7717/peerj.7434/supp-1 Supplemental Information 1 Romanov data

## Additional Information and Declarations

### Data Availability

The following information was supplied regarding data availability: Raw data is available as a Supplemental File.
